# SpaceSheep: Satellite Communications for Ovine Smart Grazing

**DOI:** 10.3390/vetsci10050340

**Published:** 2023-05-10

**Authors:** Pedro Gonçalves, Daniel Corujo

**Affiliations:** 1 Instituto de Telecomunicações, Escola Superior de Tecnologia e Gestão de Águeda, Universidade de Aveiro, 3810-193 Aveiro, Portugal; 2 Instituto de Telecomunicações, Departamento de Eletrónica, Telecomunicações e Informática, Universidade de Aveiro, 3810-193 Aveiro, Portugal; dcorujo@ua.pt

**Keywords:** animal monitoring, IoT, alarm systems, satellite communications

## Abstract

**Simple Summary:**

Animal monitoring through electronic methods requires a continuous Internet connection to guarantee timely human intervention. Internet access depends on the coverage of the communications network, which does not always exist in agricultural and rural environments, as is the case on the slopes of the Douro River. This work documents the optimization process of a monitoring and alarm generation system and its integration with a satellite communications system to guarantee alarm messages are sent in places without Internet access coverage. The results functionally demonstrated the viability of the solution for the timely delivery of alarms with an acceptable expected operating cost.

**Abstract:**

The application of IoT-based methods to support pastoralism allows the smart optimization of livestock operations and improves the efficiency of the activity. The use of autonomous animal control mechanisms frees the shepherd to carry out other tasks. However, human intervention is still needed in cases such as system failure, the bad or unpredicted behavior of the animals, or even in cases of danger, the welfare of the animal. This study documents the enhancement of an alarm generation system, initially developed within the scope of the SheepIT project, to monitor animal behavior and equipment, which warns the human operator of the occurrence of undesirable events that require intervention. Special attention was given to the use of case scenarios in places without Internet access, such as rural areas. Therefore, the system was integrated with a satellite interface, as a way of guaranteeing the timely delivery of the alarm messages. To ensure an acceptable operating cost, the system was further optimized in terms of message encoding, considering the cost of this type of communication. This study assessed the overall performance of the system, evaluated its scalability, and compared the efficiency gains from the optimization, as well as the performance of the satellite link.

## 1. Introduction

The application of ICT to support livestock activities began as a strategy to increase productivity [1,2] and reduce environmental impact. Task automation and the monitoring of the evolution of processes can reduce labor costs, allow close monitoring of electronic devices, and, consequently, impact the efficiency of livestock activities.

Within the scope of the SheepIT project [3,4,5], a postural conditioning collar for ovines was developed as an animal-based weeding mechanism, allowing the sheep to graze vineyard terrains, thereby keeping the vines and fruits safe. Sheep wear smart collars connected to an Internet of things (IoT)-like network, composed of beacons and gateways. The collars collect posture data, allowing the actuators to apply conditioning measures, whereby the gathered data is reported through the communication infrastructure. The evolution of the data was, then, analyzed using implemented analytics tools [4].

During vineyard grazing, animals are continuously tempted by vine leaves and their fruit. The collar detects the movement of the animals biting the leaves and compares the height of the neck during this process. As soon as the biting movement is detected above the maximum height threshold, the collar begins the conditioning process [3] through the emission of sound and electrostatic stimuli. Once the posture is reversed, the collar cancels the stimulation mechanism. Furthermore, so that the animals can rest at their normal size, only the biting movement triggers the execution of the posture control mechanism.

Animal behavior [6], over time, evidences the continuous attempts of the animal to violate the maximum height, while the collar avoids the animal attacks on the vine leaves and grapes. Although it has been developed as an automated conditioning mechanism, the system still requires human supervision and maintenance to ensure animal and crop safety. System failures, a lack of maintenance, and animal well-being require human supervisors to monitor animal behavior offline and intervene.

SheepIT field-trial tests [6] demonstrated the importance of automating the shepherd’s job, as labor costs were reduced by freeing the shepherd to perform other tasks in the vineyard. In addition, the use of animal monitoring tools allows the identification and prediction of a wide range of animal-related events [6,7,8,9]. In [6], Gonçalves et al. monitored the behavior of animals that were freely grazing throughout the day. It was verified that the ingestive behavior predominantly occurred at the beginning of the day, followed by a more static behavior in the afternoon, which was probably related to the digestion period. Using the same animal monitoring infrastructure, Guedes et al. [9] located the animals in a free grazing environment, having tracked the movement of the animals over a period. The results demonstrated the possibility of performing animal localization using a low-cost localization mechanism, although a localization error that depends on the location of the transmitter beacons was also found. The same monitoring infrastructure was used again, this time to create a dataset after monitoring goat kidding [7], as well as the nocturnal activity of sheep [10]. The analysis of the animals’ nocturnal activity data allowed Gonçalves et al. [8] to verify the existence of a set of rest and activity cycles during the night, as previously reported in the literature. The temporal comparison of the activity of the herd elements showed a lag in the activity of different herd members, suggesting that there may be a collective behavior of rotational herd guarding.

To optimize the shepherd’s pastoral activity and allow supervision to be conducted remotely, an event monitoring application, which kept track of the animals and the equipment was developed and integrated with the communication gateway [11]. The monitoring logic monitored the collar and beacon communications [12] and emitted an alarm whenever human supervision was required.

An alarm system depends on fast communications to operate effectively and trigger the necessary corrective measures. Such capability is not taken for granted in rural areas, where available technologies, such as a wireless local area network (WLAN) and even mobile networks [13], provide low coverage. Currently, several technologies are available that target greater coverage in IoT scenarios [14], namely NB-IoT [15], Sigfox [16], and others [17,18]. Nonetheless, these technologies are mainly only available in or around urban centers, with rural areas still left with an extensive coverage hiatus or limited access to all the technologies’ capabilities, which would be more useful in cases where IoT is used [12]. This paper documents an extension that was performed over the SheepIT system, where a satellite communications interface was added to address the difficulties in accessing the Internet, ensuring sufficient performance and availability of the alarm mechanisms.

The remainder of this paper is organized as follows: In Section 2, the proposed system is described. Section 3 assesses the communication latency and bandwidth being used, while the results are discussed in Section 4. Finally, Section 5 concludes the paper and discusses future work.

## 2. Materials and Methods

**Figure 1:**
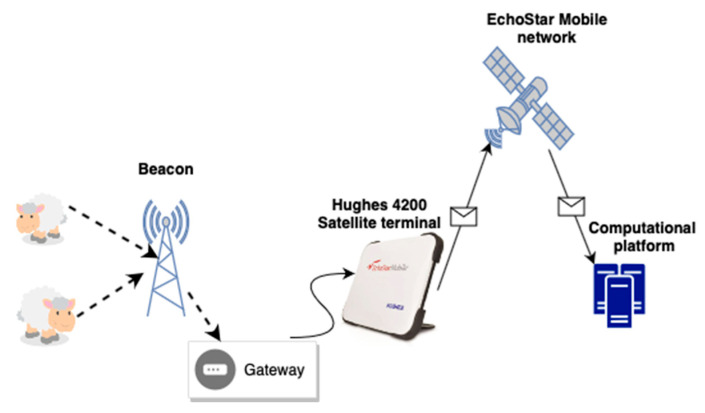
Satellite network integration.

The SheepIT collar mechanism was integrated into a wireless sensor network (WSN) [19], which was capable of monitoring the animals’ behavior [4] as well as their location [9]. The WSN, as illustrated in Figure 1, was composed of a gateway that aggregated and filtered the obtained information. It interconnects a set of fixed nodes along with a cloud-hosted system, and it can localize animals within its infrastructure. The cloud-hosted system can receive monitoring data and perform long-term analysis, assisting in determining trends, predicting behavior, and generating alarms.

### 2.1. Satellite Link Integration

The main case from SheepIT considered animal grazing in the Douro region, at Quinta da Ervamoira, an Adriano Ramos Pinto vineyard (Quinta de Ervamoira, Vila Nova de Foz Côa—41.02107689068905, —7.110593416910681), which is a place where cellular communications have very poor coverage [20], owing to the geographical rugged relief [21] that inhibits the communications between the WSN and the cloud [13]; thus, compromising the dispatching of the alarm messages. This is an ideal deployment setting for satellite-based communications [22], whereby the coverage can be provided in areas that are technically challenging or economically unfeasible for other technologies. One important characteristic of satellite-based communications is the cost per bit. Pricing models are associated with the data volume contracted by the customer; however, mobile satellite communication service providers usually place data transmission costs between EUR 4.79 and 9.58 per Mbps.

As a result, the evolution conducting the gateway of our previous solution [11], in addition to integrating and assessing a satellite communications interface that sends alarm messages, was optimized to reduce the amount of data being sent by monitoring the messages encoding optimization. In its initial form, a RabbitMQ message broker [14] producer API ensures communication between the application running in the cloud and the gateway that encodes the messages using JavaScript Object Notation (JSON) [23]. To evaluate the best high-availability and fault-tolerance solution for processing these messages, we tested Apache AVRO [24], MessagePack [25,26], and Protocol Buffers [27] for encoding the APIs.

Our testbed setup, illustrated in Figure 1, used the satellite network belonging to the EchoStar Mobile operator (Milton Keynes, UK), connected via a Hughes 4200 portable data terminal [27]. This terminal acted as a field concentrator interface for our edge computing device with the cloud service. Using a satellite link allowed the SheepIT system to be deployed in the remote unconnected zones, thereby guaranteeing smart monitoring with 24/7 operations.

### 2.2. Weeding Alarm Setup

The SheepIT alarm system was developed on the edge device because it can gather all the communications that the WSN network elements continuously send, while quickly detecting the undesired evolution in the behavior of any element of the infrastructure. The edge device continuously monitored the periodic communications related to the behavior and health of the animals as well as equipment-related events, and it detected various events that required the herd supervisor to promptly intervene, and generate an alarm.

Furthermore, both the cloud system and the edge device can generate alarms. There was a set of events that required the analysis of a larger dataset, which required computational capabilities that were not available on our edge device. Therefore, these events were detected by the cloud application. Table 1 summarizes the list of alarms triggered by the SheepIT infrastructure. One of the worst events that can occur in a SheepIT vineyard has to do with the excess of infractions detected by a collar, which should notify the human supervisor through an infracting alarm to remove the animal from the vineyard weeding job since the animal is threatening the plants/fruits.

SheepIT network nodes are battery-powered, and they periodically report their battery status to allow the system to monitor their charge and inform the supervisor about it. Collars are the key system elements because they guarantee that the animals do not harm the plants or fruits. When the battery value drops below the minimum established threshold, the system triggers an alarm to the supervisor. In addition to the batteries running out, two more types of such events can occur: equipment failure and inactivity, which can be associated with the equipment becoming inoperable, thereby freeing the animals to endanger the vine leaves. The other is identified through uncommon static accelerations that occur when the sheep has abandoned the equipment on the ground; thus, leaving the animals free to roam the vineyard.

The panic alarm is the last type of locally generated alarm. In particular, the gateway undergoes a continuous comparison between the collar accelerations and baseline acceleration values for each animal. This allows the system to detect disturbances affecting the herd, such as strangers or other animals (e.g., stray dogs).

The information sent in alarm messages, illustrated in Table 2, includes the following attributes: a timestamp with the date when the alarm was generated, the device type that identifies the type of device that triggered the alarm, the ID that identifies the device’s identifier, the alarm type, which defines the type of alarm situation, the priority level of the alarm situation, and additional information, which is an optional field with complementary information.

The cloud application, in turn, monitors the herd data and generates alarms–for example, signaling a continuous decrease in animal activity, thereby indicating a potential illness. Since these messages are generated in the cloud and are not exchanged in the satellite/WiFi links, their size was irrelevant, and therefore, their encoding was not optimized.

## 3. Results

The monitoring system was tested and functionally validated, and its performance was assessed in terms of latency and generated traffic volume. Additionally, tests were conducted using different numbers of events to determine the scalability of the system depending on the size of the herd being monitored.

To perform the tests on the system, a Raspberry PI 3 Model B+ single board computer was used to implement the gateway, and the cloud application component was hosted in a virtual machine enabled with 8 GB RAM and 2 cores, using the Ubuntu 20.04.3 LTS server Linux operating system (Ubuntu 20.04.3 LTS), which was hosted at the datacenter in the Instituto de Telecomunicações.

The performance of the signaling encoding APIs translates, especially in terms of the volume of information being transported and the latency of the communications.

### 3.1. Alarm Transfer Cost and Latency

Information transfer latency is a critical factor when it comes to an alarm transmission system. The latency of the JSON-encoded alarm system was measured and compared with the WiFi connectivity latencies. After the satellite connection was established, the messages used for testing simulated the transport of the collar information received by the WSN to the cloud-hosted application. In the first test, two metrics were used to assess the daily volume of the traffic exchanged: (a) the number of connected collars (1, 2, 5, 10, 20, 50, 100, 200, 500, and 1000); (b) the reporting periodicity (s) (30, 60, and 90). In this scenario, the same number of messages was simulated for each combination of the previous two metrics, and the duration of the experiment varied according to periodicity, with a longer duration of 90 s. From the results obtained, the amount of data generated in a 24 h interval was extrapolated, and the cost was calculated.

The results show an increase in the amount of data generated with an increase in the number of collars and a decrease in the period. The maximum volume of data was achieved for 1000 collars and a period of 30 s, with a daily volume of approximately 1.89 GB.

In terms of financial cost, considering the data transmission cost of this network (of EUR 4.79 to 9.58 per Mbps), even the unrealistic scenario, whereby the herd consisted of a single sheep, would have a significant cost: for a period of 90 s, the daily cost would be between 9.0 × 8 × 4.79 ≈ EUR 345 and 9.0 × 8 × 9.58 ≈ EUR 690.

Regarding the second test, the latency of the system for the transmission of collar information was registered at different times during the day, using both WiFi connectivity and EchoStar’s satellite link. This experiment was repeated 10 times for both 1 and 100 collars. The results were registered with a 95% confidence interval using a t-distribution.

**Figure 2:**
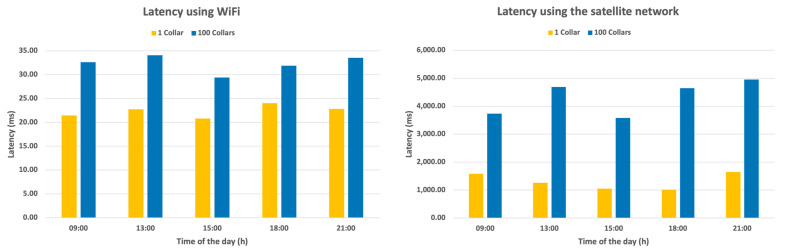
System alarm dispatch latency for several hours of WiFi network (**left**) and satellite network (**right**).

As for the results test, Figure 2 shows the variation in the time taken to send the information. As can be seen, in both cases the latency increased alongside the message size. Both technologies showed some fluctuations throughout the day, although this would have no impact when applied in a realistic scenario.

### 3.2. Alarm Encoding Evaluation

The alarm encoding evaluation focuses on the assessment of three serialization formats: Apache Avro, MessagePack, and Protocol Buffers. For each existing type of alarm, a varied number of messages were generated through an alarm simulator created in the gateway. The maximum number of messages simulated for the two types of devices (collars and beacons) was different because a real system uses a reduced number of beacons to cover the pasture area while monitoring a herd that is typically composed of a greater number of animals.

**Figure 3:**
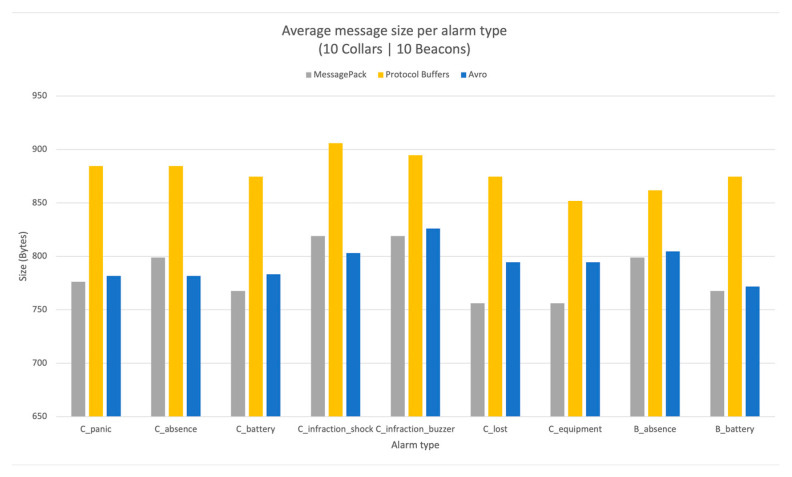
Analysis of the size of generated information for different alarms and encoding APIs.

The tests were repeated 10 times for each combination, and the average was registered with a 95% confidence interval using a t-distribution. The performance of the encoding APIs was measured with respect to the message size through a network sniffer. Figure 3 demonstrates the encoding message sizes for each of the alarm messages supported by the system in the three encoding APIs under investigation.

The test in the previous section revealed that using MessagePack is an adequate format to encode the information forwarded to the cloud-hosted application. Moreover, experiences show that the evolution of the system’s latency in alarm message transmission using the different APIs follows the encoding efficiency. As a result, the alarm types that produce larger messages also take longer to transfer information.

Once the greater efficiency of the MessagePack API was verified, the JSON encoding API was replaced by the MessagePack API, and the tests for transferring gateway-generated alarms were repeated. To assess the impact of these changes, two metrics were measured: the volume of the traffic produced, and the corresponding latency.

**Figure 4:**
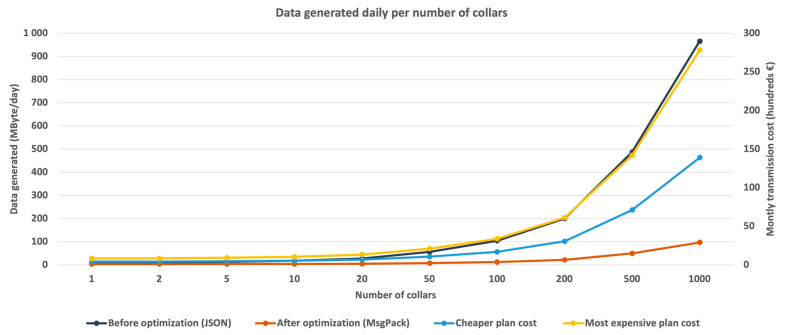
Comparison between the original JSON format and MessagePack encoding efficiencies.

The test conditions were similar to those of the initial tests in terms of the number of alarms and the distribution of alarm types, yet the periodicity messaging was set to 60 s. These results were compared to the results obtained before the system optimization and are presented in Figure 4. Unsurprisingly, the alarm dispatching latency followed the message volume for both encodings.

The monthly costs associated with the transmission, considering the transmission price for one MB, are represented in Figure 4 as hundreds of euros per month, and grow, as expected, alongside the size of the herd being monitored.

### 3.3. On-Site Tests

**Figure 5:**
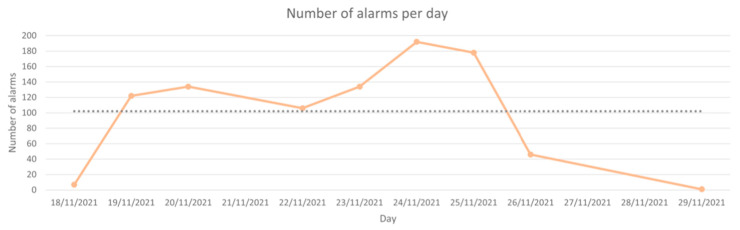
Variation in the number of alarms throughout the 12 days.

To test the performance of the system in a real environment, we simulated sending events recorded by the gateway during the monitoring period for a flock of 18 sheep between November 18th and 29th 2021; Figure 5 shows the number of alarms that were generated for the days observed. Since most alarms are associated with the behavior of living animals, the number of alarms generated daily does not follow any regular pattern. Furthermore, the number of alarms reached its maximum value on the 24th of November, when 192 alarms were detected. Approximately 44% of the daily values were in the range of 100 and 140, while 22% of the days generated fewer than 10 alarms. The system registered an average of approximately 100 alarms per day.

Table 3 displays the number of alarms and their sizes produced during those days, which are grouped by alarm type. Some alarm types have greater values than expected (in the realistic scenario), which are explained by the characteristics of the system in question: (i) the infraction alarms were produced despite the conditioning being disabled since the tests were carried out in November after the vines were harvested; thus, the animals were not warned when committing an undesirable behavior, thereby continued to perform the same actions and triggered their alarms repeatedly; (ii) the beacon did not cover the entire pasture area, which meant that some of the animals were not detected, even though they were inside the designated area.

To validate the possibility that the hardware being used in the gateway did not compromise performance, the alarm detection and sending times were measured, both for the gateway implemented on the Raspberry Pi and on the Core i7 4500U (1.8 GHz), 16 GB RAM PC.

**Figure 6:**
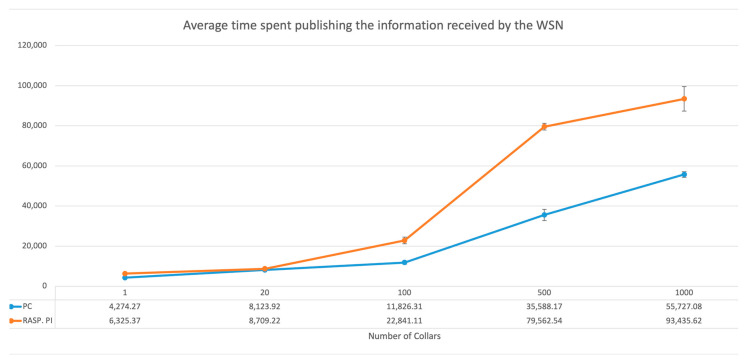
Impact of the number of collars on the time spent publishing the information received by the WSN.

As for the temporal analysis results illustrated in Figure 6, the computer presented a better performance than the Raspberry Pi in every test, which was expected due to its higher processing capacity.

## 4. Discussion

The results of the first test showed an increase in the amount of data generated with an increase in the number of collars and a decrease in the period (Figure 2), with a daily volume of 1.89 GB for 1000 collars and a period of 30 s. Although this volume of information can be transferred by technology, which can transfer up to 2.99 GB daily, it is quite expensive, with daily values between EUR 345 and 690. These considerably expensive values would be even greater for larger herds, as they are directly proportional to the values of the generated data, as shown in Figure 2. As such, an increase in the data transfer period is not a viable solution, since the volume of data produced continues to represent a great expense for the system; therefore, message encoding optimization is required.

The results of the connection latency test evidence a higher value for the satellite case, which was associated with the increase in the size of the messages. When smaller messages are considered, the latency is also affected by the different data paths being used by the two technologies. Thus, when larger messages are used, the overall latency reached one and a half seconds, which is a much higher latency than that measured on the WiFi connection, especially due to the low bandwidth available on the satellite connection. In fact, the satellite connection presented a greater latency than the WiFi because of two major factors. Firstly, the path taken by the messages was not the same for each technology. The information sent via the satellite link travels to the destination via a greater number of hops; 23 hops were verified for the satellite connection and 9 when using the WiFi connection. Finally, the data rate available for the satellite connection was limited, and thus, the same information took more time to be uploaded in the case of this connection. Nevertheless, the satellite communications latencies cannot be considered a limiting factor for the system.

The encoding performance comparison results shown in Figure 3 consistently demonstrate the higher efficiency of the MessagePack API in carrying the alarm messages. AVRO encoding performed second in the tests, and Protocol Buffers had the worst performance in the tests. The difference between MessagePack and Avro was systematically small, while that between MessagePack and Protocol Buffers was slightly larger, with an approximately 8% difference.

The signaling traffic volume generated by the system, before and after message encoding optimization, decreased considerably and allowed a significant reduction in the amount of information exchanged daily, with approximately 10 times less data from 100 collars upwards. Considering a tariff based on the information volume, the optimization has a considerable impact on both the costs and system latency. The modification of encoding the messages created a significant reduction in the amount of information exchanged daily, with approximately 10 times less data from 100 collars upwards. In terms of latency, it reached 4 s when the maximum number of collars was tested, while for 100 collars, it decreased by approximately 2.9 times.

In terms of transmission costs and considering tariffs based on the volume of data transmitted, the savings were significant, albeit insufficient. Considering a herd of 100 elements, the expected monthly cost of transmission would decrease from approximately EUR 14,000 per month to EUR 1680, which remains financially unaffordable.

The analysis of the computational requirements of the alarm system showed that for small and medium-sized herds, the hardware currently running at the gateway (Raspberry PI) responds to the set of requests imposed by system events; however, for larger herds, with more than 100 animals, the response times grow too large to allow for timely alarms to be sent. Unsurprisingly, in the case of the gateway implemented via a PC, the times grew much slower, which made it possible to create a gateway solution with better performance for larger herds.

## 5. Conclusions

In addition to grazing in the vineyard, animal conditioning mechanisms have been used with other species, such as olive groves, orchards, and even blueberry plantations. Likewise, its application capabilities can go beyond animal conditioning, with wearable sensing devices allowing the monitoring of feeding, time of ingestion accounting, the studying of the energy expended by animal activities, deliveries, and even night activity and wellbeing. In most of these locations, where such activities are common, Internet access is limited, as was the case at Quinta da Ervamoira. Nonetheless, electronic systems still depend on the action of the human operator in certain situations, such as when the equipment fails or when the well-being of the animal is at stake.

The present work allowed for the implementation of a monitoring system for animal devices, which is able to send an alarm that helps to protect the vineyards and animals. SheepIT gateway was integrated with a satellite interface, allowing it to operate even in places without the Internet, such as areas near the Douro River region in Portugal. In addition to integration with a satellite communications system, the present work included the evaluation of several information-encoding APIs to optimize the sending of information related to the alarms, and the replacement of the JSON API, which was initially used by the MessagePack API.

The monitoring system was functionally validated and the communication latency was evaluated, while the volume of the signaling produced during its operation was measured by different encoding APIs. The signaling measurements revealed that MessagePack performed better. Moreover, the results showed that the performance values were perfectly acceptable and compatible with the system’s alarm function.

Additionally, the analysis of the computational requirements necessary for the implementation of the gateway showed that for small/medium-sized herds, it is possible to maintain a gateway based on a Raspberry Pi, yet for larger-sized herds, it will be necessary to improve the computational capacity of the hardware that implements the gateway.

In any future work, the set of alarms being transmitted via satellites should be minimized, as well as any alternatives for sending more efficient messages. A LoRaWAN-satellite integrated environment should be considered, which is supported by an AI-assisted opportunistic transmission approach that can allow timely alarm transmissions, and opportunistically transmit low-cost monitoring data for behavior analyses through data mining techniques.

## Figures and Tables

**Table 1:** System alarms.

Type of Alarm	Alarm Source	Alarm Context	Message Size (Bytes)
Battery	Gateway	Network node (collar/beacon) battery level below a minimum threshold	1134
Absence	Gateway	Network node (collar/beacon) no longer detected after several communication cycles	1147
Infraction	Gateway	Number of infractions per unit of time crossed the predefined threshold by an animal	1161
Panic	Gateway	Abnormal accelerations from multiple elements of the herd were detected in the same period	1159
Inactivity	Gateway	Detection of a pattern of collar inactivity, evidencing that the animal may have removed the collar.	1134
Health	Cloud	Prolonged decrease in an animal’s activity was detected	-

**Table 2:** Examples of alarm notifications.

Timestamp	Device Type	ID	Alarm Type	Priority	Additional Information
Sat Nov 20 07:22:36 2021	Beacon	1	Battery	Low	Battery: 18 (%)
Sat Nov 20 07:36:12 2021	Collar	2	Infraction	Low	#Warnings = 21 (in 3 min)
Sat Nov 20 08:19:47 2021	Beacon	1	Battery	High	Battery: 6 (%)
Sat Nov 20 08:37:14 2021	Collar	2	Equipment	High	

**Table 3:** Number of alarms generated for alarm type.

Device	Alarm Type		Size (Bytes)
Collar	Panic	6	1159
	Absence	75	1147
	Battery	2	1137
	Infraction	689	1126
	Lost	0	1134
	Equipment	141	1157
Beacon	Absence	3	1159
	Battery	4	1134

## Data Availability

Not applicable.
